# High-resolution in-depth imaging of optically cleared thick samples using an adaptive SPIM

**DOI:** 10.1038/srep16898

**Published:** 2015-11-18

**Authors:** Aurore Masson, Paul Escande, Céline Frongia, Grégory Clouvel, Bernard Ducommun, Corinne Lorenzo

**Affiliations:** 1 Université de Toulouse, ITAV-USR3505, F-31106 Toulouse, France; 2 CNRS, ITAV-USR3505, F-31106 Toulouse, France; 3 ISAE, DISC, F-31106 Toulouse, France; 4 CHU de Toulouse, F-31059 Toulouse, France; 5 Imagine Optic Orsay F-91400, France

## Abstract

Today, Light Sheet Fluorescence Microscopy (LSFM) makes it possible to image fluorescent samples through depths of several hundreds of microns. However, LSFM also suffers from scattering, absorption and optical aberrations. Spatial variations in the refractive index inside the samples cause major changes to the light path resulting in loss of signal and contrast in the deepest regions, thus impairing in-depth imaging capability. These effects are particularly marked when inhomogeneous, complex biological samples are under study. Recently, chemical treatments have been developed to render a sample transparent by homogenizing its refractive index (RI), consequently enabling a reduction of scattering phenomena and a simplification of optical aberration patterns. One drawback of these methods is that the resulting RI of cleared samples does not match the working RI medium generally used for LSFM lenses. This RI mismatch leads to the presence of low-order aberrations and therefore to a significant degradation of image quality. In this paper, we introduce an original optical-chemical combined method based on an adaptive SPIM and a water-based clearing protocol enabling compensation for aberrations arising from RI mismatches induced by optical clearing methods and acquisition of high-resolution in-depth images of optically cleared complex thick samples such as Multi-Cellular Tumour Spheroids.

Light sheet fluorescence microscopy (LSFM), also known as Selective plane illumination microscopy (SPIM), represents a universal and versatile technique for three-dimensional (3D) imaging of live tissues and organisms with subcellular resolution^1^,^2^,^3^,^4^ and, undoubtedly, is emerging as a useful tool for performing 3D imaging of complex thick biological samples^3^,^5^. Nevertheless, as for all fluorescence microscopes, it still remains limited for in-depth imaging of scattering and of heterogeneous samples. Indeed, optical aberrations, absorption and scattering of both excitation and emission result in a loss of signal and contrast, limiting practical use for imaging up to a few hundred μm deep. In complex thick samples, scattering and optical aberrations arising from refractive index (RI) discontinuities between and within cells are the main processes which contribute to degradation of image quality^6^ and which limit the resolving power of optical imaging techniques. To overcome these obstacles, LSFM can be combined with an optical clearing method which chemically treats tissues to render them transparent^7^,^8^,^9^,^10^,^11^. Recent progress in tissue clearing methods has facilitated microscopic analysis of whole embryos, tissues and intact organisms. These methods work by minimizing RI mismatches in tissues so that photons undergo less, or almost no, scattering. Furthermore, by homogenizing the RI in fixed samples, optical aberrations induced by the sample itself are reduced or eliminated.

However, achieving high transparency in the sample is not enough to acquire high-resolution 3D images. Indeed, a common problem in imaging optically cleared samples is the immersion media of objectives. The latter are designed to work with a specific RI medium (*n*_*1*_) that often differs from the RI of the clearing agent (*n*_*2*_), therefore leading to a mismatch between RI *n_1_* and *n_2_*. Consequently, strong defocus and spherical aberrations are imparted into the system, causing significant light perturbation such as defocus and focal elongation, together with a significant decrease in signal-to-noise ratio and resolution^12^. Furthermore, aberration increases in proportion to imaging depth, thus hindering high-resolution imaging of large volumetric samples. Recently, however, the availability of customised objectives specifically designed to work with given clearing agents has made it possible to solve this problem^13^. But since their development is still in its infancy, few such affordable objectives are on the market. An alternative solution has been suggested by Silvestri & co-worker^14^, consisting of correcting aberrations by means of adaptive optics (AO). A method originally developed for astronomic telescopes, AO measures and corrects aberration, thus restoring optimum performance of the imaging system^15^. Over the past decade, AO has been implemented in microscopy^16^ and has proven to be a valuable tool for correcting aberration and restoring diffraction-limited resolution in different kinds of fluorescence microscopes^17^,^18^,^19^,^20^. Recently, we successfully applied a Wavefront sensor Adaptive Optics scheme to use with SPIM (_WAO_SPIM) to correct complex aberration induced by the sample itself^21^. We demonstrated the ability of _WAO_SPIM to improve in-depth imaging of deep complex samples such as Multi Cellular Tumour Spheroids (MCTS). These are tissue-mimic models that are highly useful in investigating the influence of malignant cell interactions during cell proliferation^22^. Due to their opacity and density, however, they raise significant challenges for imaging by light microscopy. We have shown that spatial variation of aberrations within such thick, non-transparent biological samples causes major changes to the light path and limits the quality of AO correction in an extended field of view^21^.

To date, AO has not yet been combined with optical clearing imaging methods. In the current study, we propose a straightforward method for aberration correction relying on the use of _WAO_SPIM in an open-loop mode and on a single set of corrections, enabling us to improve imaging across the entire volume of cleared samples studied. Furthermore, we demonstrate that this method combined with deconvolution helps recover more details and suffer less artifacts. This efficient, user-friendly and versatile strategy which makes it possible to compensate for aberrations arising from RI mismatches induced by optical clearing methods was applied to 3D imaging of cleared complex thick tissue mimics represented here by MCTS.

## Results

### The source of aberration: theoretical analysis

**Figure 1:**
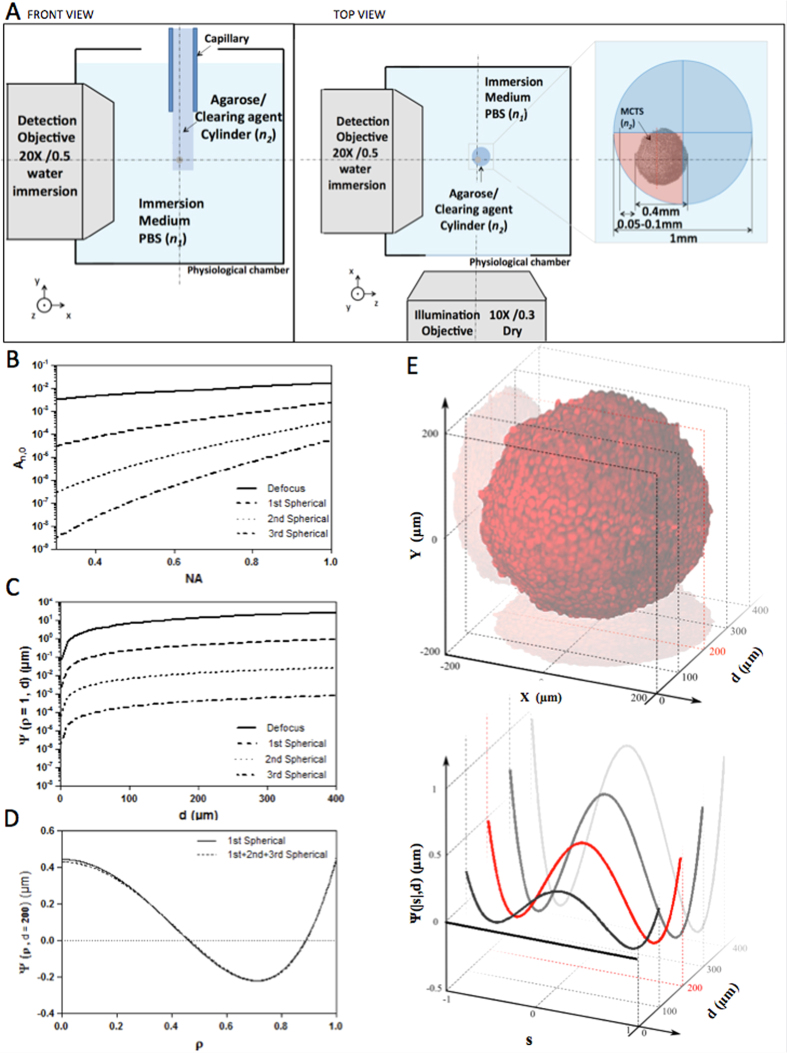
(**A**) Schematic drawing (top view and side view) of the SPIM physiological chamber and the sample-mounting configuration with a zoom view of the geometry of agarose/clearing agent cylinder in which the MCTS was included. (**B**) Magnitude of aberration coefficients Eqs (3 and 4) as a function of NA. (**C**,**D**) Aberration function Ψ*(ρ,d)* as function of *d* for *ρ* = 1 (**B**) or as function of *ρ* for *d* = 200 μm (**D**) Eq (2). Different aberration terms Ψ calculated for an axial distance *d* = 200 μm considering only the 1st spherical order (d, solid line) and composed by the sum of the first three spherical terms (d, dashed line). (**E**) Evolution of Ψ for different *d* values. The edge of MCTS was estimated to be at *d* = 50 μm. The red line corresponds to the compensated optical section (*d* = 200 μm), where average amplitude of Ψ is predicted.

To test the proposed method, MCTS either labelled with the EdU Click chemistry method (MCTS_EdU) and enabling DNA detection in proliferating cells or MCTS stably expressing a histone H2B nuclear protein (H2B) fused to the mCherry fluorescent protein (MCTS_mCherryH2B) were cleared by using the most recently developed method, CUBIC^23^. CUBIC is a water-based optical clearing method that works by (1) breaking down lipids in fixed tissues and (2) replacing the surrounding intracellular media by a higher RI solution. In our system, detection was performed using water-dipping objective lenses. The sample was embedded in a cylinder with a mixture of 2% agarose and clearing agent that was immersed in PBS. CUBIC is a reversible method, which means that after several hours of incubation in PBS/water, cleared MCTS nearly fully recovered their appearance. Thus, embedding the cleared MCTS in a mixture of agarose and clearing agent enabled us to efficiently clear the sample and to maintain the sample clarity by limiting reversibility in PBS/water. A schematic illustration of the sample mounting geometry is shown in Fig. 1A. Applying CUBIC led to a refractive index mismatch between the clearing agent (RI: 1.48–1.49, Fig. 1A) and the objective immersion medium (herein RI: 1.33, Fig. 1A), thereby introducing defocus and spherical aberration. This delayed a fraction of ballistic photons and contributed to point spread function (PSF) enlargement and therefore to resolution degradation.

Defocus and spherical aberrations are well known^24^,^25^ and can be understood by considering the shape of the wavefront in the pupil plane (P) of an objective lens with a given RI *n*_*1*_. Since optical clearing methods result in RI homogenisation within the sample, we assumed the cleared MCTS to be a homogeneous medium of uniform RI *n*_*2*._ In a first approximation, we also assumed that the interface between the mismatched mediums was flat. In this case, the pupil function was modelled by


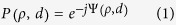


where 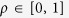 is the normalized pupil radius and *d* is the nominal focusing depth in a perfectly matched system (absence of a RI boundary). In an aberrated system, the pupil function is modified by the wave aberration function Ψ (or phase error). This function can be decomposed as a weighted sum of Zernike polynomials (Z) and can be expanded into a series of radially symmetric Zernike polynomials of zero azimuthal order (Z_*j,i*_, where *j* and *i* are respectively the radial and azimuthal orders) with aberration coefficients *A*_*j,0*_


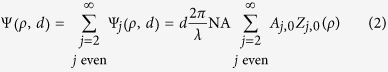


Here *j* represents the orders of defocus (*j* = 2) and all higher order spherical aberrations (*j* = 4, 6, 8, …), NA is the numerical aperture, and *λ* the fluorescence emission wavelength. Considering only defocus and spherical aberrations, the aberration coefficients can be calculated using eqs (3) and (4)


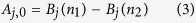


where


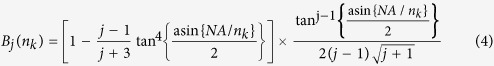


The numerical computation of these equations shows the strong influence of the NA objective, the nominal focusing depth, and the RI mismatch on the extent of defocus and spherical aberration (Fig. 1B–D). The aberration coefficients A_*j,0*_ were plotted as a function of NA, for *j* = 2, 4, 6 and 8 (Fig. 1B). As shown, aberration coefficients A_*j,0*_ are dominated by defocus (*j* = 2) and 1^-st^ order spherical aberration (*j* = 4) and drop for high orders of *j*. High NA objectives are more sensitive to spherical aberrations than low NA objectives. Considering the detection objective (X20, NA 0.5) and the emission wavelength used in this study (510 nm), we calculated the corresponding aberration function (Ψ_*j*_(ρ,d)) (Fig. 1C). The latter rises proportionally to the nominal focusing depth corresponding to the distance between the edge of the sample and the depth of the focal plane. In this current optical configuration, a nominal focusing depth of 400 μm, for example, produced a focusing error of 50 μm (Supplementary Fig. S1a,b). Second and 3^rd^ spherical orders for an objective with a NA of 0.5 can be disregarded (respectively 10^−6^ and 10^−7^, Fig. 1B). However, the addition of oscillating terms, even low-amplitude ones such as spherical aberrations, might induce a major wavefront modification. For this reason, it was important to ensure that the sum of the 1^-st^, 2^nd^ and 3^rd^ spherical orders gave rise to the same result as when considering only the 1^-st^ order spherical aberration (Fig. 1D). We calculated the root mean square error (RMSE) to compare the difference between the aberration function Ψ corresponding to the sum of the first 3 spherical orders (*j* = 4,6,8) and the aberration function relative to the 1^-st^ spherical order (*j* = 4).


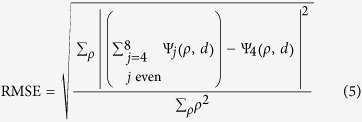


This deviation was estimated at 0.023%, meaning that using a model considering only the 1^-st^ spherical order was well adapted in our case.

From the numerical simulation, we observed that the amplitude of the aberration function increased proportionally with depth and yielded an intermediate weight contribution at around 200 μm deep within the cleared MCTS (Fig. 1E). This imaging plane, hereafter known as the compensated optical section, corresponded to the MCTS centre minus the distance (usually 50–100 μm) between the immersion medium (*n*_*1*_)/mounting cylinder (*n*_*2*_) interface and the cleared MCTS boundary (Fig. 1A). The distance variation was essentially due to the sample mounting procedure. The samples were most often found to be located in one of the cylinder quarters, rarely centered (Fig. 1A). This current depth-dependent spherical aberration model assumes a flat interface between the mismatched mediums. It is well known that a cylindrical-shaped sample holder (Fig. 1A) introduces new aberrations, which must also be taken into consideration. As described by Bourgenot and colleagues^26^, the primary source of aberrations resulting from the cylindrical interface with a sample holder is astigmatism. In addition, it has been shown by Turaga and Holy^27^ that the magnitude of aberrations such as astigmatism, coma and trefoil, arising by imaging at a tilt through a planar surface between mediums of mismatched refractive index, increased with the tilt angle. In this particular case, the detection axis was not perpendicular to the sample interface and the rays travelling in the propagation direction were subject to different sample thicknesses. A similar effect occurs with cylindrical interfaces and lead to the same nature of aberration pattern. However, in our case the sample position could not be precisely controlled inside the agarose/clearing agent cylinder, thus leading to—high variability of these aberration magnitudes.

## Experimental Results

**Figure 2:**
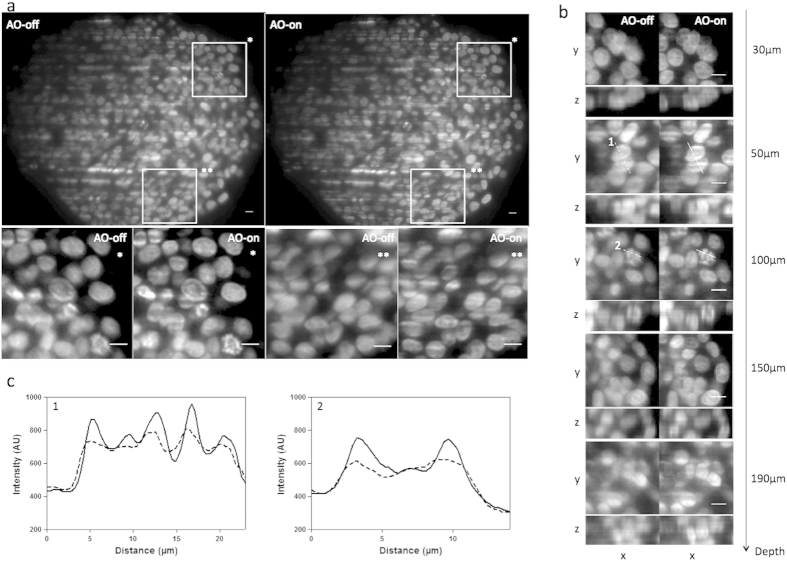
Cleared MCTS labelled with EDU-click Alexa 594 images were acquired with _WAO_SPIM either without (AO-off) or with (AO-on) correction at a fixed excitation intensity at 594 nm and a 200 ms exposure time. Light sheet illumination comes from the right. (**a**) Top: cleared MCTS-EdU images acquired at a focal depth of 100 μm before (AO-off) and after (AO-on) aberration correction. Bottom: Magnified views of top inset views (*and **). Scale bar 10 μm. (**b**) Subregions of (AO-off) and (AO-on) images taken at different focal depths (30, 50, 100, 150, 190 μm). Scale Bar 10 μm. (**c**) Intensity profiles along lines 1 (50 μm in depth) and 2 (100 μm in depth) indicated in (**b**). Scale bar = 10 μm.

In order to minimize the aberrations due to sample positioning, it was important 

to systematically position the samples in the same way in the _WAO_SPIM. Therefore, sample holders were carefully rotated until the quarter cylinder where the MCTS was closest to the cylinder edge was located in front of the objective lens (Fig. 1A). After that, the light sheet was refocused manually on the cleared MCTS compensated optical section. This step, consisting of translating the light sheet in the *z* axis (approximately 1–2 μm) and in the *x* axis (approximately 6.5 ± 2.5 μm, mean ± SD) until obtaining a clear image, enabled us to compensate for focus error (Supplementary Fig. S1c). Then, in order to achieve high-resolution images of cleared MCTS, aberration correction was performed by using an open-loop strategy, which consisted in first manually adjusting spherical aberration and defocus (residual errors) and then the other modes of higher amplitude accountable for major phase errors such as astigmatism, coma and trefoil. Prior to this step the deformable mirror command matrix was computed during a calibration process based on the characterisation of each actuator mirror using a reference sample (see Methods). Table 1 gives the list of Zernike terms with their corresponding equation used for the subsequent experiments (indices given by the supplier). The first 10 Zernike azimuthal orders were used, excluding the lowest two values corresponding to tilts. The latter did not affect the image quality. Overall, the user successively adjusted the coefficient value for each Zernike mode with reference to the compensated optical section image output until a clear image with maximum possible brightness and sharpness levels was obtained. This procedure can easily be performed in the same way that objective correction collars can be adjusted. Once the set of Zernike coefficients was adjusted and applied a *z*-stack of the entire cleared MCTS was then acquired either with or without correction (respectively, AO-on or AO-off) and compared. Supplementary Figure S2 shows the Zernike coefficients of two individual sample corrections and the average magnitude of Zernike terms corrected for different experiments. As expected, the RI mismatch introduced spherical aberrations. Results show that predicted values of spherical aberrations (0.18 μm) were close to the experimental data (0.10 μm ± 0.05, mean ± SD) (Supplementary Fig. S2). We also observed that defocus was almost fully compensated for after light sheet refocusing (residual focus error 0.02 μm ± 0.01, mean ± SD). In addition, we also encountered astigmatism, coma and trefoil errors. Although the samples were carefully positioned in the same way for each experiment, the magnitude of these aberrations was significantly different from one sample to other (Supplementary Fig. S2) and was thus difficult to predict. In terms of image quality, the corrected images displayed improved sharpness and attenuated blur (Fig. 2; Supplementary Movie S1). The gain in information was clearly illustrated by the apparition of thin structures unresolved without correction. Moreover, improvement was visible throughout all the optical section images of cleared MCTS (Fig. 2A,B). The apparition of structural details could be seen in the first planes (30 μm in depth) and beyond 100 μm deep to the compensated optical section (190 μm in depth) (Fig. 2B). In order to qualitatively evaluate improvement in image quality after correction, fluorescence intensity profiles along different lines were extracted from Fig. 2B and analysed (Fig. 2C). After correction (AO-on), we could clearly distinguish distinctly separated and higher intensity peaks in the profiles of both lines (1) and (2) corresponding, respectively, to condensed DNA of a prometaphase figure and to poorly resolved nuclei distinguishable without correction (AO-off).

### Image quality assessment

**Figure 3:**
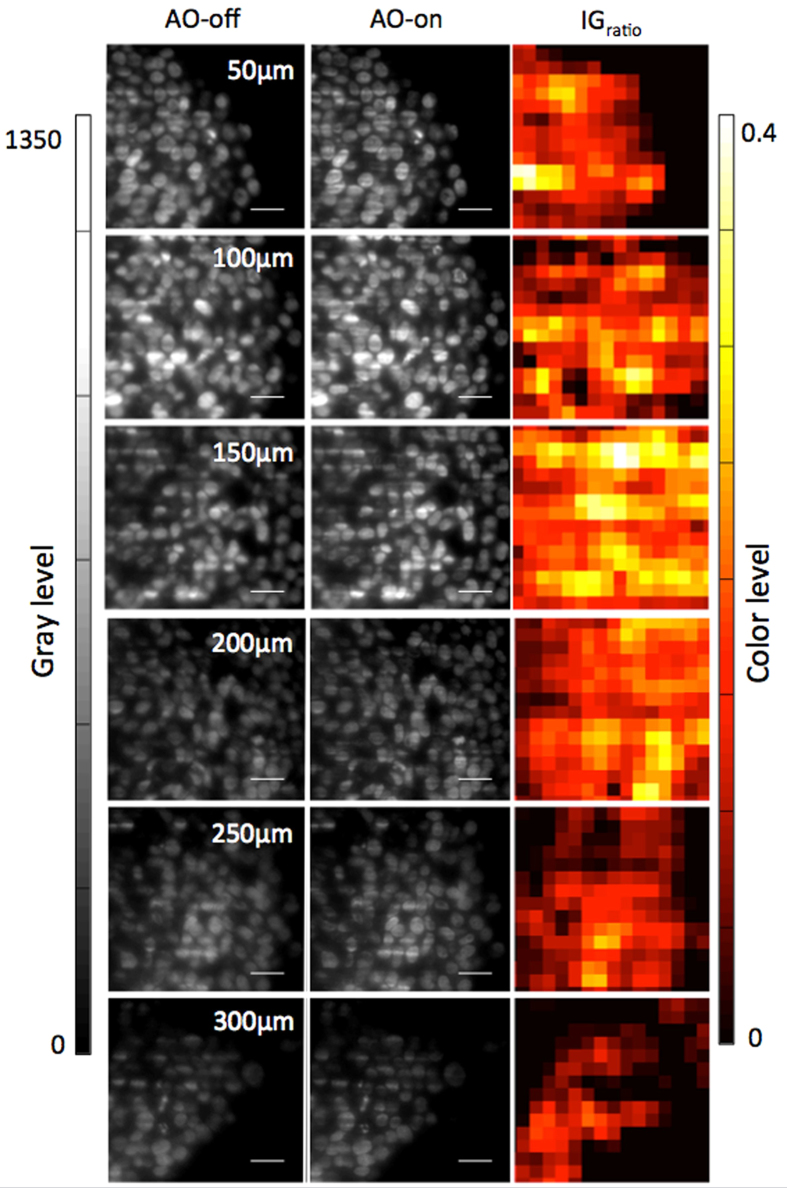
***IG***_***ROI***_**Ratio of cleared MCTS_EdU image of subregions taken at different focal depths (50, 100, 150, 200, 250, 300 μm), calculated as** 
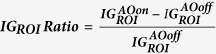
 **where** 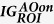 **is** 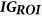 **mapping image calculated from images obtained with correction and** 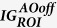 **is** 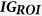 **mapping image calculated from images obtained without correction.** The square size of ROI corresponds to a typical diameter of a nucleus (10 μm). Scale bar = 25 μm.

In addition to fluorescence intensity profiles, improvement of image quality was then also quantitatively evaluated in terms of local contrast and spatial frequencies. The former were measured according to Jorand *et al*.^21^, using a gradient intensity map representing the local total variance computed for square Regions Of Interest (ROIs). Intensity gradient map ratios (IG_ratio_) of different optical sections of cleared MCTS_EdU are shown in Fig. 3. The AO-on images represent those acquired at different focal depths after performing aberration correction on the compensated optical section. Local contrast enhancement can be clearly seen for all optical section images of cleared MCTS_EdU. We measured an average improvement of 10–20%, as high as 40% in some subregions (Fig. 3). Up to 200 μm in depth, correction was uniformly efficient across the field of view. Beyond 250 μm in depth, contrast improvement was less noticeable than in the first optical section images of cleared MCTS. As expected, correction was more efficient and uniformly close to the compensated optical section (located in this case at 150 μm deep), but nonetheless, a significant improvement (approximately 10%) was maintained even at a depth of 300 μm.

To get an accurate assessment of the improvement provided by correction, we decided to introduce a novel metric based on spatial frequencies allowing us to quantify and compare each spatial frequency component of images which were corrected or not. The aim was to evaluate the gain in spatial resolution for each object size in the image. Since spatial frequency components carry information about the image in great detail the more contrasted the latter become after aberration correction, the more the frequency weight should increase. A ratio factor was calculated corresponding to the relative difference of each frequency (RD) by comparing each component to the reference measured in the AO-off image.

For an 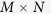
image *u* its 2-D discrete Fourier Transform was defined as follows:


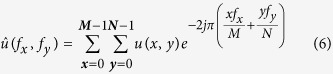


with 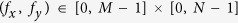 being the coordinates in the frequency domain. This representation enabled us to localize the spatial frequency corresponding to each object with a specific size defined by *d*_*r*_. The latter was radially spread out around the zero frequency and was distributed in a ring 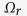 which comprised |*f*_*x*_| = *M*/2*d*_*r*_ in the *x* direction and |*f*_*y*_| = *N*/2*d*_*r*_ in the *y* direction. Spatial frequency components contained in each ring 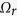 were summed to create a radial component associated to the radial frequency (*f*_*r*_). We constructed 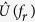, the normalized radial representation of 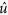



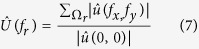


To evaluate the improvement of images, we then calculated the relative differences (RD) of radial frequency between images acquired before, 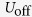, and after, 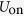, correction. RD was defined by


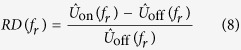


**Figure 4:**
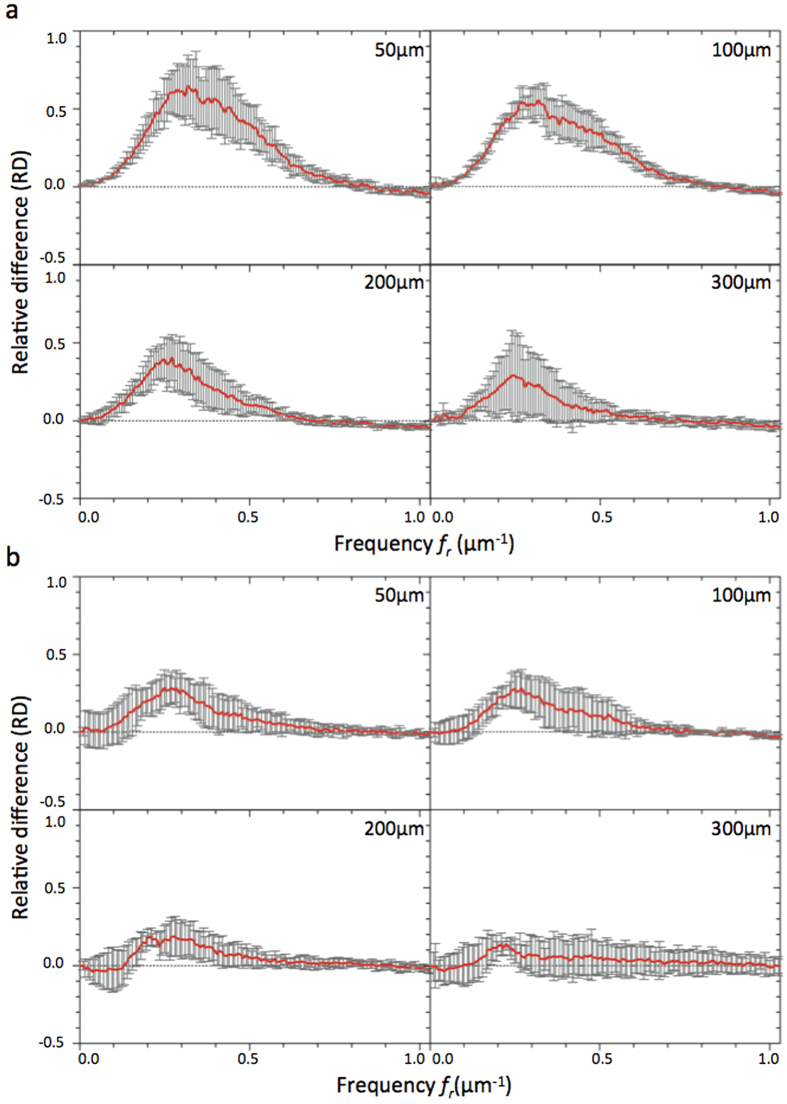
RD curves corresponding to the relative difference between Fourier contents of AO-on and AO-off images obtained for different optical section images of cleared MCTS_EdU (**a**) and cleared MCTS_mCherryH2B (**b**) immersed for 5 days in CUBIC reagent 1. Mean curves are estimated from n = 4 experiments and error bars represent the standard deviation.

**Figure 5:**
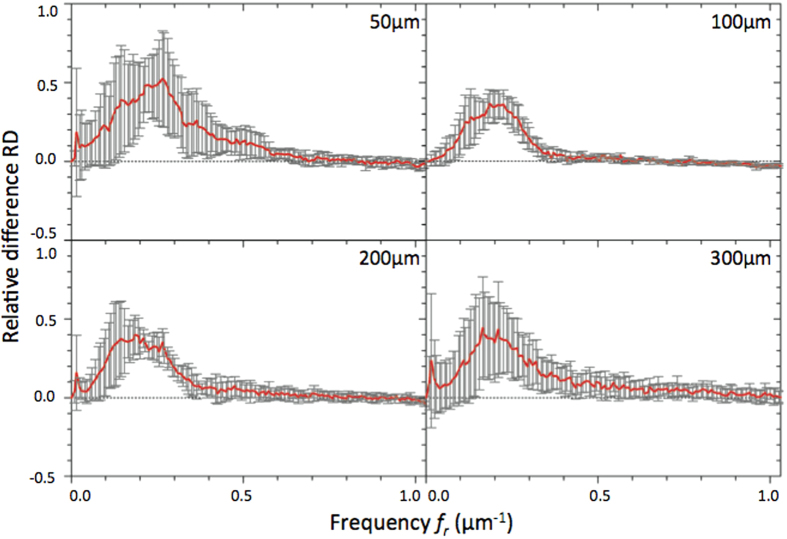
RD curves corresponding to the relative difference between Fourier contents of AO-on and AO-off images obtained for different optical section images of cleared MCTS_mCherryH2B immersed for 2 days in CUBIC reagent 1. Mean curves are estimated from n = 4 experiments, and error bars represent standard deviation.

RD values were plotted in function of spatial frequencies (Figs 4 and 5) for different optical section images of cleared MCTS_EdU (Fig. 4A) and cleared MCTS_mCherryH2B (Figs 4B and 5). The maximum value of spatial frequencies referred to in *x* coordinates (1.03 μm^−1^) corresponds to the frequency cut-off of our optical system (resolving power limit). Consequently, values above this limit had no significance and are not reported. Positive values of RD indicated an effective correction for a given frequency (*f*_*r*_) thus enabling us to retrieve certain details characterized by specific size, while negative RD values indicated an inefficient correction leading instead to degradation of information. Regardless of the imaging depth, we observed a similar distribution of RD with a peak for spatial frequencies ranging from 0.25 to 0.35 μm^−1^ and a full width at half maximum for frequencies ranging from 0.15 to 0.55 μm^−1,^ corresponding to object size typically matching that of nuclei and subnuclear structures (ranging from 6.7 to 1.8 μm). Maximal values of RD were found for the optical section images acquired in the first 100 microns and decreased beyond that up to a factor two. Interestingly, we noted that the frequency weight varied less (standard deviation) around the compensated optical section (100–150 μm in depth), indicating that image quality enhancement was robust around the compensated optical section and somewhat less beyond. Furthermore, we observed weaker values of RD and greater variability for cleared MCTS_mCherryH2B (Fig. 4B) than for cleared MCTS_EdU (Fig. 4A). Although the enhancement in image quality was still quantifiable, its efficiency was noticeably lower. Fluorescence preservation and clearing processes are known to be important trade-offs since the chemical treatments generally used in clearing methods increase transparency but at the same time degrade fluorescence of proteinaceous fluorophores such as mCherry. CUBIC is well known for preserving fluorescence in this context, but we nonetheless observed a drop in mCherry fluorescence levels after 5 days with the first CUBIC reagent (R1) which led to a low level of fluorescence. This was verified by reducing R1 incubation time to 2 days in order to better preserve the mCherry fluorescence (Fig. 5). This change, however, was accompanied by inefficiently cleared processes. Maximum RD values were higher (0.5 *vs*. 0.3) in this case than for cleared MCTS_ mCherryH2B incubated for 5 days in R1 and were equivalent to those obtained for cleared MCTS_EdU. Nevertheless, the correction was less reliable (greater variability), and the improvement in image quality was observable only within a restricted range of frequencies, from 0.1 to 0.3 μm^−1^. Finally, despite weak signal intensity levels, which could have been a limiting factor for correction, we were still able to achieve a certain degree of correction and thus significantly improve images of cleared MCTS_mCherryH2B. The signal intensity level must therefore be recognized as an important factor in correction ability and, consequently, in achieving high image quality.

### AO improve the deconvolution process

**Figure 6:**
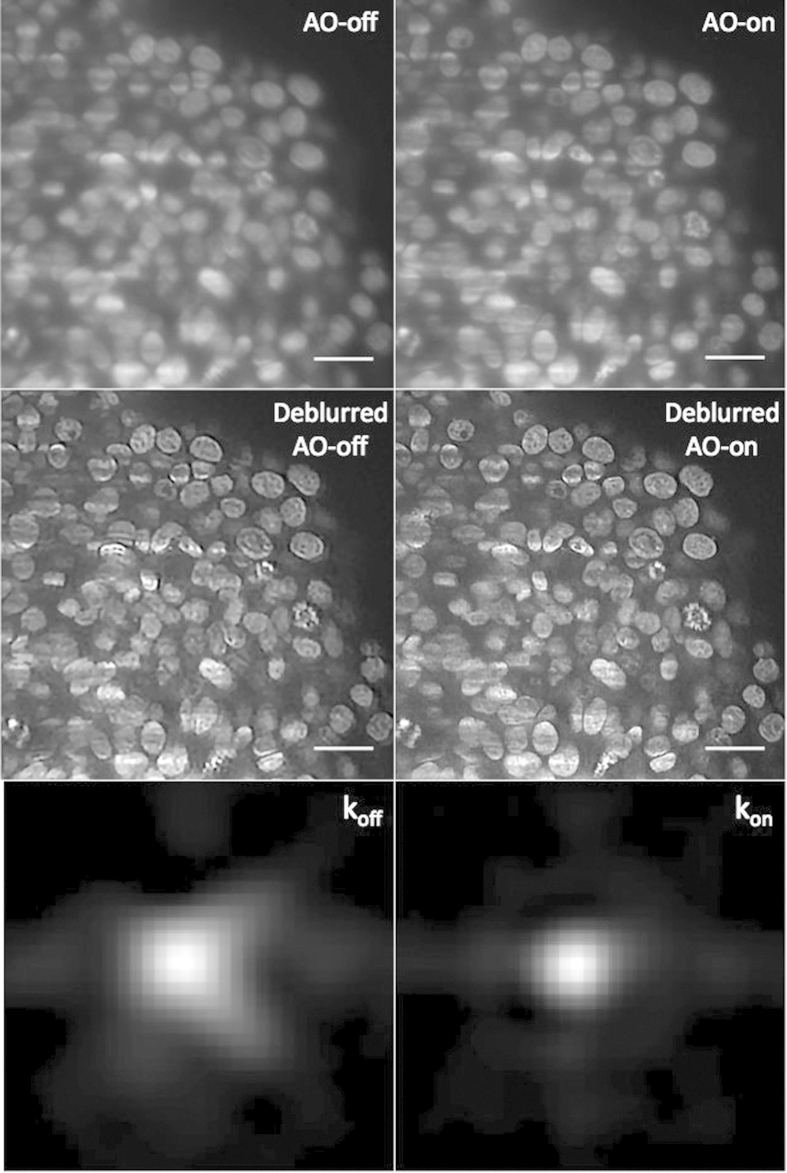
Images of cleared MCTS_EdU images (corresponding to the compensated optical section, depth = 100 μm) acquired with (AO-on) or without (AO-off) correction and obtained after blind deconvolution. k_off_ and k_on_ represent the PSF function between the deblurred and the raw AO-off and AO-on images calculated by the deconvolution algorithm. Scale bar = 25 μm.

To deconvolve the images, we used blind-image deconvolution making it possible to recover a sharp image by researching the unknown kernel k blur^28^ (Supplementary information S1). Figure 6 shows the resulting deconvolved images acquired with (AO-on) or without (AO-off) correction, which we called respectively “Deblurred AO-on” and “Deblurred AO-off”. In the case of an initially sharp image, this algorithm makes it possible to expect an estimated PSF tending to a Dirac function. We observed that for the uncorrected images, the corresponding deblurred images were enhanced with artificially added high frequencies. Furthermore, they were associated with a larger kernel. In contrast, deblurred AO-on images were well defined. As shown in Supplementary Figure S3 and Movie S2, improvements could be observed in the whole deblurred AO-on *z*-stack images compared to deblurred AO-off sample. In conclusion, image correction enabled us to reveal details not visible without correction and contributed to successful deconvolution since the deconvolution process was not efficient on uncorrected images. Consequently, this regularisation approach can be effectively combined with correction in order to obtain a final sharp deblurred image.

### Versatility of the method

To further demonstrate the versatility of our strategy and its compatibility with any water-based clearing method and any kind of staining, we then used the CLARITY method^29^,^30^ with our samples. This method, compatible with immunostaining applications, is based on transforming tissue to a nanoporous hydrogel and removing lipids with an active or passive diffusion process based on ionic detergent solubilisation. MCTS were cleared with CLARITY (RI of approximately 1.45) and subsequently immunostained with an antibody directed against the α tubulin protein, a constituent of microtubule structure. We chose this particular structure in order to test correction ability in sparsely labelled samples. In contrast to CUBIC, CLARITY is an irreversible method making it possible to immerse the cleared samples directly in water/PBS while maintaining their transparency. Therefore the cleared MCTS were embedded in a cylinder of 1% agarose which was then immersed in PBS. In this case, the RI of sample mounting holders was close to water, which enabled us to disregard aberration arising from sample positioning. Indeed astigmatism, coma and trefoil terms were not present and only defocus and spherical aberration had to be compensated for to improve image quality (Supplementary Fig. S4a). Supplementary Figure S4 shows the resulting images of microtubules, corrected (AO-on) or not (AO-off), with the corresponding local contrast map (IG) and RD plots. The microtubules were imaged at a depth of approximately 60 μm. After correction, contrast improvement was significant and enabled us to clearly distinguish particular fine structures such as the cytokinetic bridge (Supplementary Fig. S4b) and the mitotic spindle (Supplementary Fig. S4c).

## Discussion

SPIM has emerged as a versatile and indispensable instrument for long-term live 3D imaging of organism models. Furthermore, recent progress in optical clearing methods and their combination with LSFM have, without any doubt, improved optical in-depth imaging capabilities in large-scale tissue samples. Our results show that it is indeed possible to simplify the aberration pattern of tissue mimics, such as MCTS, by optically clearing them. In this context we chose water-based clearing methods such as, for example, CUBIC or CLARITY for their compatibility with SPIM configuration^10^. However, even if these methods guarantee high transparency and simplify MCTS aberration patterns, it was apparent that even a small RI mismatch between immersion medium and cleared samples can cause a significant loss of resolution due to spherical aberrations. RI mismatch is thus a key consideration for high-resolution in-depth imaging of water-based cleared samples. However, the correction procedure is greatly simplified in this context since the optical properties of the sample can be approximated, thus enabling predictive correction. This fact motivated us to exploit the potential of _WAO_SPIM for correcting this kind of aberration.

To address this issue, we first provided a comprehensive and simple model based on RI mismatch in _WAO_SPIM. The numerical simulation study of phase errors showed that low-order aberration correction driven by the radially symmetric Zernike terms should be sufficient to provide significant image quality improvement. We then defined a single focal depth, the compensated optical section, where the contribution weight of phase errors was of an intermediate level, and where we subsequently carried out the open loop. However, depending on the geometry and nature of the sample mounting holder, sample-positioning aberrations such as astigmatism, coma and trefoil can be found and must be taken into consideration. Our results closely reflected the simulation, and it was experimentally demonstrated that correcting from 4 to 10 Zernike terms provides significant recovery of signal and resolution. In addition, we showed that even for small initial aberrations, low-order correction is still beneficial. Image quality improvement was evaluated using two image quality metrics based, respectively, on gradient intensity and spatial frequency content. In general, the corrected images show considerably improved quality in terms of peak intensities and contrast. However, we were not able to reveal image quality improvement for cleared MCTS_mCherryH2B by using the gradient intensity map, whereas the spatial frequencies content metric did reflect significant enhancement. In other words, the intensity gradient map does not provide consistently reliable information on images with low signal-to-noise ratio such as cleared MCTS_mCherryH2B images. In contrast, RD is suitable when signal-to-noise ratio is low. Overall, our results demonstrate that this “semi-empirical “ strategy is robust with respect to changes in experimental conditions. In particular, it makes it possible to reach a satisfactory compromise in image quality improvement over all focal depths and can also be easily used in different kinds of staining and clearing protocols. Finally, we have shown that this method, when combined with fast space-invariant deconvolution algorithms, improves the quality of raw corrected images and ensures that the PSF is closer to the theoretical model.

However, when imaging at different depths, our strategy does not currently provide plane-by-plane correction. An approach using an image-based sensorless adaptive scheme^31^ would merit exploration in the future on, for example, (i) methods relying on the theoretical model described above (Supplementary information S2), or (ii) iterative algorithms based on trial-and-error to optimally converge within an image quality metric.

In conclusion, we propose a simple and user-friendly procedure enabling image quality improvement in light sheet microscopy for cleared samples capable of dealing with all existing and forthcoming water-based clearing methods. With the rapidly growing list of clearing protocols, there has never been a greater need for solutions making it possible to compensate for a range of RI for different kinds of cleared samples. Ongoing advances in the combination of these methods with sophisticated techniques such as _WAO_SPIM significantly contribute to a better understanding of the practical benefits and the trade-offs of optical-chemical combined methods, and will undoubtedly open up new experimental avenues for exploring thick complex samples such as tissue mimics.

## Methods

### _WAO_SPIM setup

The layout of the set-up is shown in Supplementary Figure S6. The illumination path (orange and blue lines) is similar to that of a conventional SPIM. The main difference concerns the detection path (red and green lines) where an adaptive optic loop has been implemented. For more details see reference^21^.

### Calibration procedure

The AO loop correction was driven by CASAO^TM^ software (Imagine Optic) which enables communication with the Hartman Shack Wavefront sensor (HSWF) and the Deformable Mirror (DM). The calibration procedure began by positioning a fluorescent bead in the field of view. An interaction matrix was then computed between HSWF and DM. In the algorithm implemented in the CASAO^TM^ software, information from the HSWF sensor is decomposed into the orthogonal modes of the DM using a standard algorithm of singular value decomposition. A command matrix was then calculated making it possible to correct up to 40 modes. Prior to the calibration procedure, the “static” aberrations due to the non-common path between the HSWF and the camera were measured and were compensated for. The purpose of this step was to set the DM shape so that it would correct imaging path aberrations. The corresponding DM shape was referred to as “AO-off ”. For more details see reference^21^.

### Cell culture

HCT116 wild type and HCT116-mCherry_H2B cells were cultured in DMEM + GlutaMAX (Dulbecco’s Modified Eagle Medium; Gibco), supplemented with 10% fetal bovine serum and 1% penicillin–streptomycin (Pen Strep; Gibson) and maintained at 37 °C with 5% CO_2_ in an incubator.

### MCTS production

To produce the MCTS, we used the centrifugation method described in^3^ . MCTS were prepared in 96-well plates that were coated with 20 mg/ml polyHEMA (Sigma). Cells were plated at a density of 600 cells/well in 100 μl cell culture medium per well then centrifuged to enable MCTS formation. After 4–5 days of growth, MCTS of 400 μm in diameter were collected, washed three times with PBS and then fixed with 10% neutral buffered formalin (Sigma–Aldrich) at room temperature over 2 hours.

### EdU labelling of MCTS

EdU labelling was performed by using the Click EdU Alexa Fluor imaging kit (Molecular Probes). Briefly, EdU (5-ethynyl-2 ´-deoxyuridine) is a thymidine analogue which can be incorporated into the DNA during the DNA synthesis phase (phase S) in proliferating cells. EdU detection relies on a simple and quick click reaction, a copper-catalyzed covalent reaction between an azide and an alkyne, that does not necessitate a DNA denaturation step such asBrdU detection. EdU was added to the culture medium to obtain a final concentration of 10 μM. After 24 hours of incubation at 37 °C, MCTS were washed in PBS and then fixed. EdU detection, based on the click reaction between EdU and Alexa Fluor 594 dye, was performed following the manufacturer’s instructions.

### Immunofluorescence

Following the clearing protocol, immunohistological studies can be successfully carried out by allowing for penetration of biomolecules. Cleared-MCTS were washed in PBS/0.5% BSA/0.1% Triton v/v. Incubation was carried out with antibodies against αTubulin (mouse polyclonal, Sigma, 1/2000 at 4 °C, for 4 days). After being washed in PBS/0.1% Triton v/v, the secondary antibody was added (anti-mouse conjugated with Alexa594, Molecular Probes, 1/800, at room temperature, over a 4-day period.

### CUBIC protocol

CUBIC (Clear, Unobstructed Brain Imaging Cocktail) is a chemical reversible method adapted for clearing large structure like mouse organs^9^. Two main reagents are used: Reagent 1 (R1) was prepared as a mixture of 25 wt% urea (U5378, Sigma Aldrich) 25 wt% N,N,N′,N′-tetrakis (2 hydroxypropyl) ethylenediamine (122262, Sigma Aldrich) and 15 wt% polyethylene glycol mono-ρ-isooctylphenyl ether/Triton X-100 (2000, EUROMEDEX). Reagent 2 (R2) was prepared with 50 wt% sucrose, 25 wt% urea, 10 wt% 2,2′,2″-nitrilotriethanol (90279, Sigma Aldrich) and 0.1 v/v% Triton X-100 (2000, EUROMEDEX).

For MCTS clearing, samples were immersed in R1 at 37 °C with gentle shaking for either 2 or 4 days, after which the solution was changed and the sample immersed in the same volume of fresh R1 for an additional 3–4 days. Then different washing steps were carried out at room temperature to gently adapt the refractive index inside the MCTS: 1) with a PBS solution, several times, 2) with a PBS/sucrose 20% solution for a minimum of 2 hours, and 3) with a PBS/glycerol 50% solution for a minimum of 2 hours. MCTS were then immersed in R2 at 37 °C with gentle shaking for 1–2 days. For storage, the samples were placed in PBS at 4 °C after R1 step. Washing and R2 immersion steps were pursued before imaging experiments. For _WAO_SPIM imaging, CUBIC-cleared MCTS were embedded in a cylinder made of a mixture of low-melting 2% agarose and clearing R2 agent (usually one third for two thirds of final volume).

### CLARITY protocol

CLARITY is an irreversible clearing technology developed around non-reversible chemical transformation^29^. This method brings transparency and permeability to macromolecules by removing lipids. Biological samples are transformed into a stable macromolecule-permeable structure. MCTS are embedded for 4 days at 4 °C in a hydrogel mixture of 3% Acrylamide /BisAcrylamide (A4058, Sigma Aldrich), 0.15% N,N,N′,N′-Tetramethylethylene-diamine (T9281, Sigma Aldrich), 0.007% Ammonium persulfate (Sigma Aldrich), 3.6% Formaldehyde (F8775, Sigma Aldrich). The second solution is the clearing reagent made with 4% SDS (Ambion), boric acid (Sigma Aldrich), with sufficient H_2_0, to reach PH 8.5 NaOH (Sigma Aldrich). After a polymerization step of at least 3 hours at 43 °C, the clearing solution was added and maintained for 7 days at 43 °C. CLARITY-cleared MCTS can be stored at 4 °C in PBS. Transparency of the MCTS was sufficiently stable in PBS to either embedded them in a 1% agarose cylinder or to put them into a sample chamber inside a phytagel container filled with PBS as described in Desmaison *& al.*, 2012.

### Imaging processing

Images were processed with the open-source image-processing Fiji package and with MATLAB. SPIM images were registered with the StackReg plugin available in the Fiji software. For image quality assessment (spatial frequency comparison and intensity gradient maps), Matlab codes were developed and are available on request.

## Additional Information

**How to cite this article**: Masson, A. *et al*. High-resolution in-depth imaging of optically cleared thick samples using an adaptive SPIM. *Sci. Rep*. **5**, 16898; doi: 10.1038/srep16898 (2015).

## Supplementary Material

Supplementary Information

Supplementary Movie S1

Supplementary Movie S2

## Figures and Tables

**Table 1:** List of the Zernike terms with their corresponding equation used in this study.

Haso index	Equation	Name
3	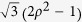	Focus
4	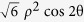	Astigmatism 0°
5	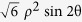	Astigmatism 45°
6	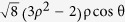	Coma 0°
7	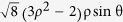	Coma 45°
8	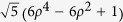	3rd order Spherical
9	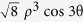	Trefoil 0°
10	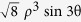	Trefoil 30°
