# Supplementary material for: High-resolution in-depth imaging of optically cleared thick samples using an adaptive SPIM
Source: Sci Rep. 2015 Nov 18;5:16898. doi: 10.1038/srep16898 (PMC4649629; doi:10.1038/srep16898)
Supplement: Supplementary Information [file srep16898-s1.pdf]

High-resolution in-depth imaging of optically cleared thick samples using an adaptive SPIM

Aurore MASSON[1,2], Paul ESCANDE[1,2], Céline FRONGIA[1,2], Grégory CLOUVEL[4],Bernard DUCOMMUN[1,2,3] and Corinne LORENZO[1,2].

Supplementary Figure S1

(a) Schematic representation of refraction rays at the sample interface n_1/n_2. The axial distance d_1 is the nominal focal position of the objective lens, d_2 is the focusing depth in the n_2 medium, and the difference $\delta d = d_2 - d_1$ is the focusing error. (b) Focusing error curve δd is represented as a function of d_1. (c) Fixed MCTS_EdU or cleared MCTS_EdU acquired both with $_{WAO}$SPIM at different depths (right and centered panels). Cleared MCTS_EdU images acquired with $_{WAO}$SPIM after refocusing the light sheet illumination (left panels). These images as referred as AO-off. Scale bar 25μm.

Supplementary Figure S2

(a, b) RMS value of Zernike coefficients applied for two individual AO experiments and (c) RMS values average of Zernike coefficients used for different experiments (n=5). The error bars represent the standard deviation.

Supplementary Figure S3

Cleared MCTS_EdU images obtained by blind deconvolution at different depths taken with (AO-on) or without (AO-off) correction. Scale bar 25μm.

Supplementary Figure S4

$_{WAO}$SPIM correction (AO-on mode) of MCTS cleared with the CLARITY protocol and immunostained with an antibody directed against alpha tubulin. (a) RMS values of Zernike coefficients applied for this experiment. (b) and (c) corresponds to different regions within the MCTS acquired at 60μm in depth respectively with (AO-on) or without (AO-off) correction. Intensity gradient map (IG off, IG on) and relative difference of the spatial frequencies for (a) and (b) are represented. Scale bar = 10 μm.

Supplementary Figure S5

Experimental set-up picture of the $_{\text{WAO}}$SPIM.

Supplementary data S1

Blind image deconvolution is a very ill-posed problem where both the PSF and the sharpen image have to be estimated. Many deconvolution methods have been proposed when the PSF was known[1-4]. Algorithms based on Richardson-Lucy are probably the more common in the microscopy community. However, they are highly sensitive to PSF estimation errors, which lead to strong ringing artifacts in the restored image. Therefore more robust methods are essential to tackle the problem of the joint estimation of the PSF and the sharpen image. In this work, we used one of the state-of-the-art methods in blind deconvolution [5] based on the minimization of a cost function derived using advanced regularization terms. This robust and efficient method offers an estimation of the PSF and a deblurred image.

Supplementary data S2

The numerical simulation based on eq(2) shows that each independant aberrating function Ψ_n increases linearly with depth. In perspectives, we can build a dynamic model which take account the depth progression during the z-stack image acquisition. Until now, coefficient values are entered in the CASAO software and SPIM image acquisition are done with a home-made program developed on the Labview platform (National Instrument). An implementation of synchronization steps can be made in that software to communicate with CASAO software and automatically increment coefficient values depending on the image position.

Supplementary Movie S1

Z stack images (step1μm) of cleared MCTS_EdU acquired with AO--on or AO-off.

Supplementary Movie S2

Z stack images (step1μm) of deblurred cleared MCTS_EdU images acquired with AO-on or AO-off.

Supplementary References

1. Richarson, W.H. Bayesian-Based Iterative Method of Image Restoration. J. Opt. Soc. Am. 62, 55-59 (1972).
2. Hansen, P.C., Nagy, J. G., & O'leary, D. P. Deblurring images: matrices, spectra and filtering. SIAM 3 (2006)
3. Chan, T.F.C.K.W. Total variation blind deconvolution. Image Processing, IEEE Transactions 7, 370-375 (1998).
4. Daubechies, I., & Teschke, G. Variational image restoration by means of wavelets: Simultaneous decomposition, deblurring, and denoising. Applied and Computational Harmonic Analysis 19, 1-16 (2005).
5. Krishnan, D., Tay, T., & Fergus, R. Blind deconvolution using a normalized sparsity measure. Computer Vision and Pattern Recognition 2011 IEEE Conference, 233-240 (2011).

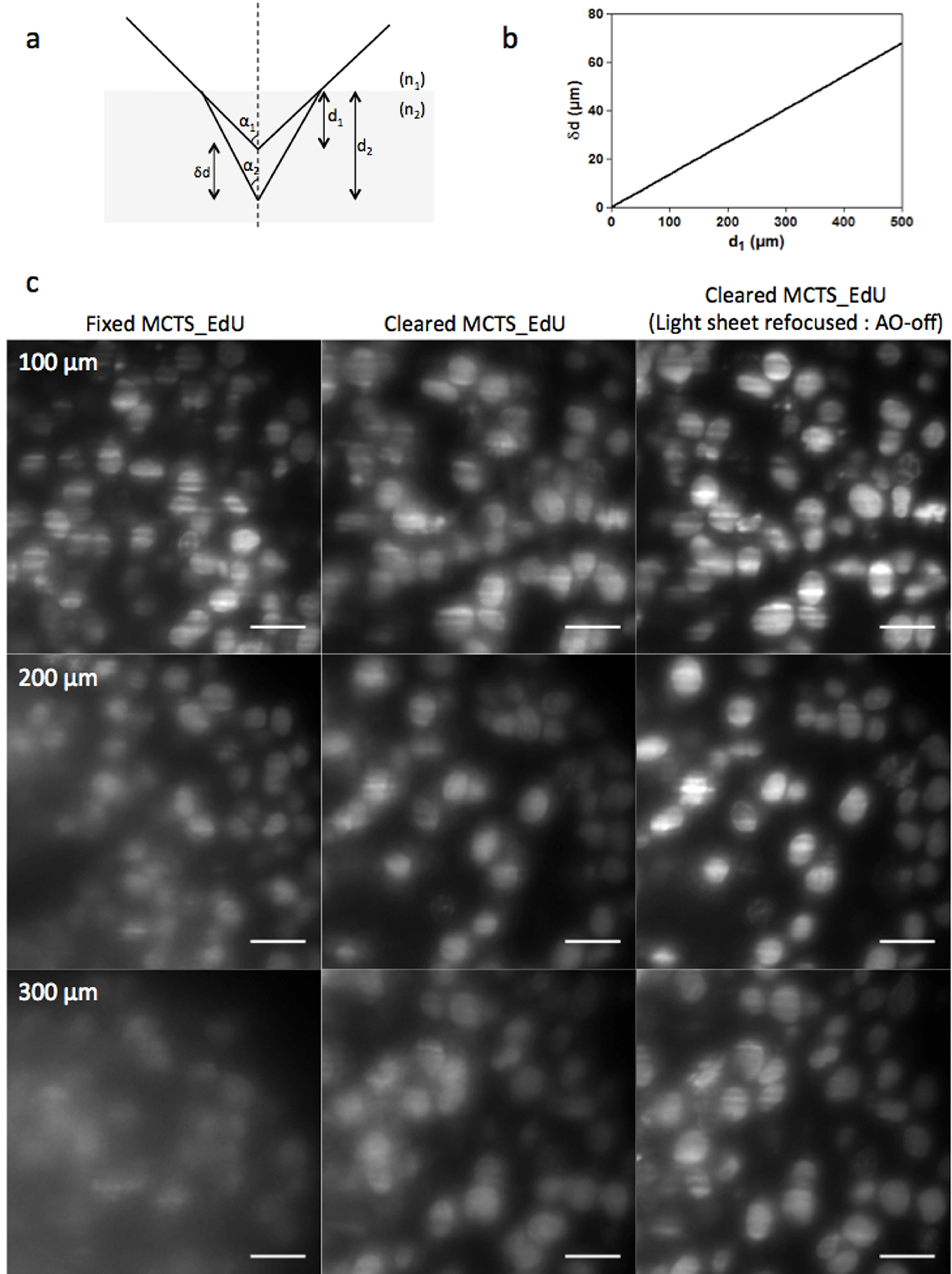

a
(n1)
(n2)
α1
α2
δd
d1
d2
b
δd (µm)
d1 (µm)
c
Fixed MCTS_EdU
Cleared MCTS_EdU
Cleared MCTS_EdU
(Light sheet refocused : AO-off)
100 µm
200 µm
300 µm

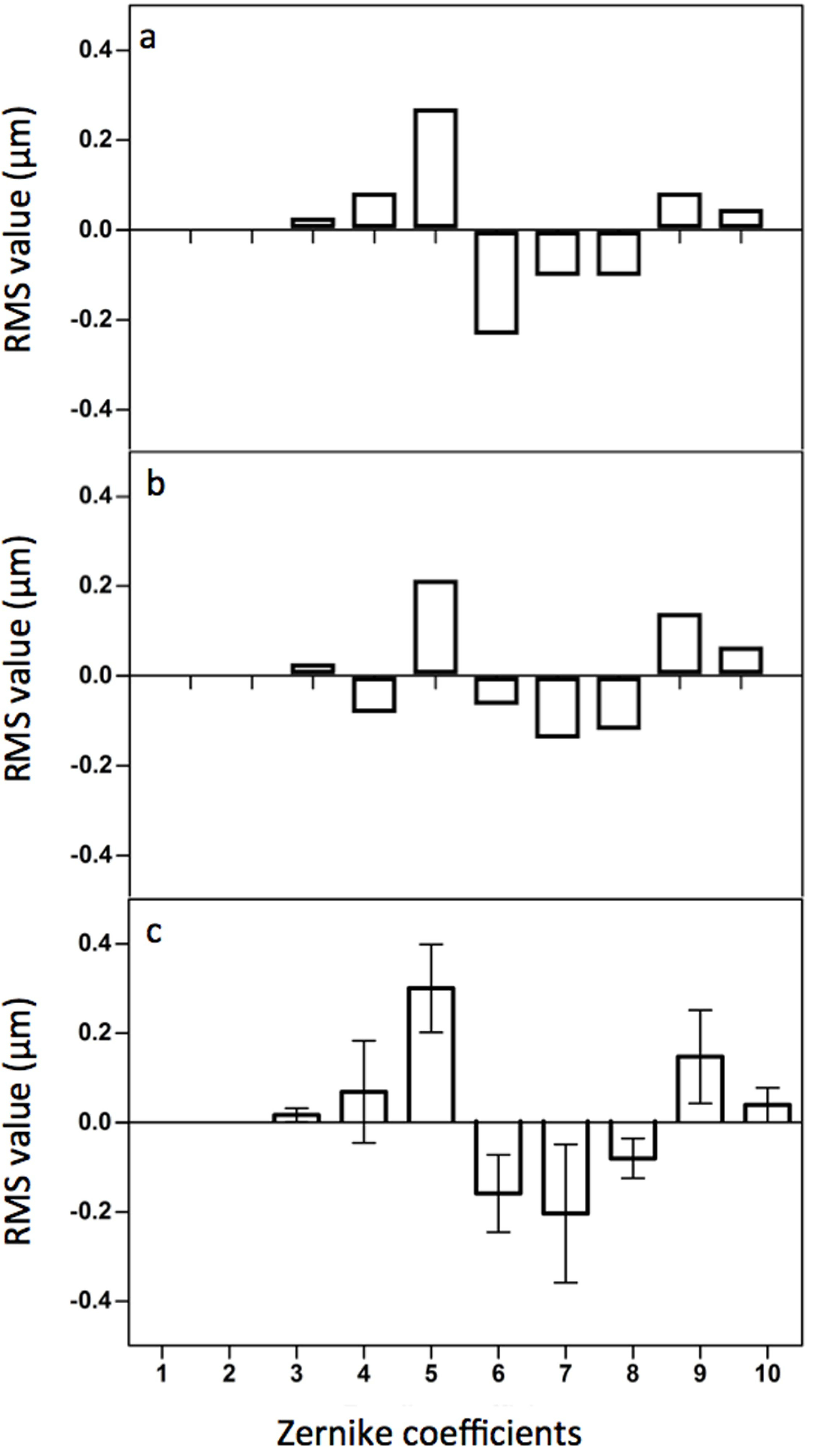
a
b
c
0.4
0.2
0.0
-0.2
-0.4
RMS value (µm)
1
2
3
4
5
6
7
8
9
10
Zernike coefficients

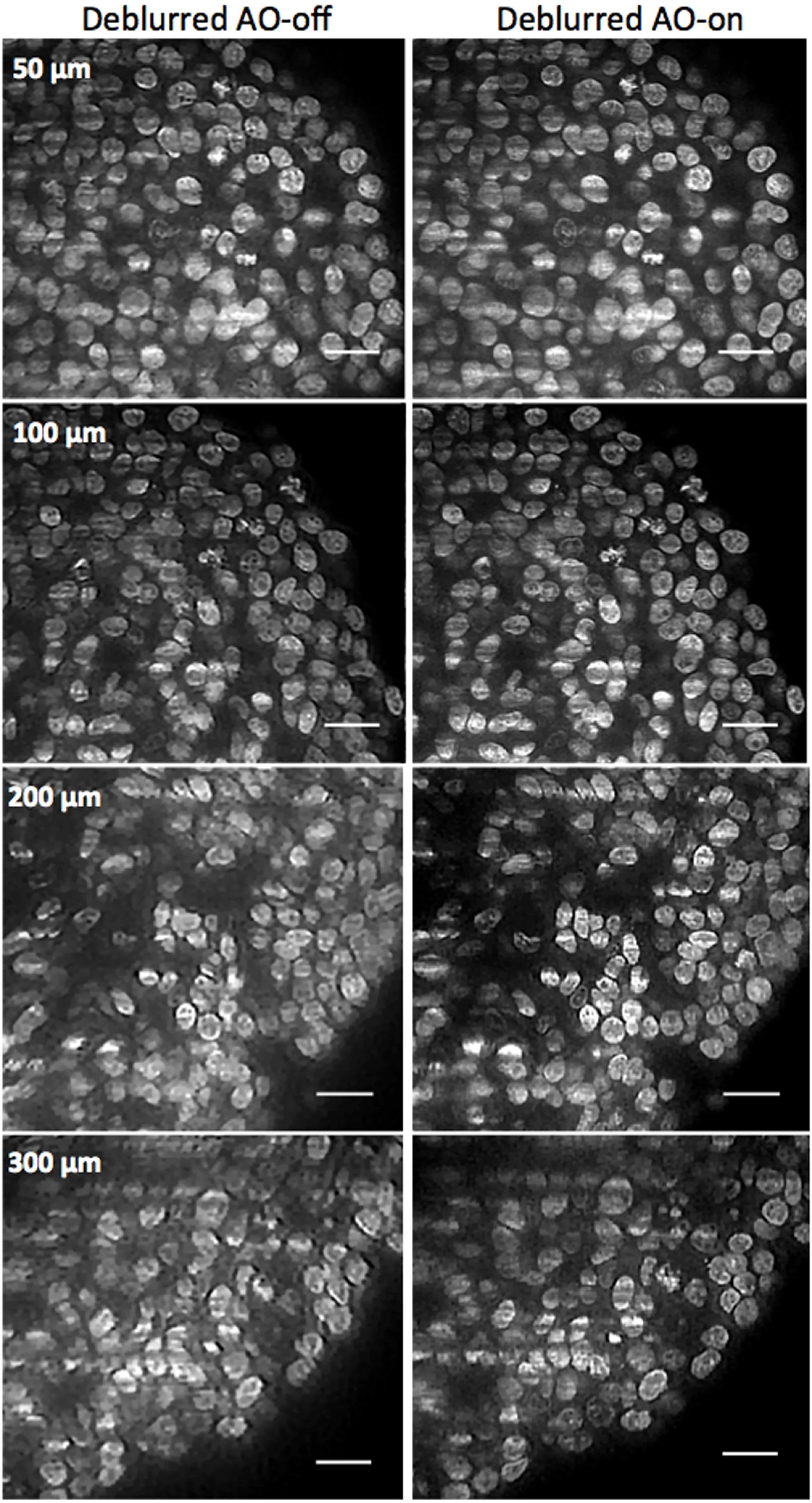
Deblurred AO-off
Deblurred AO-on
50 µm
100 µm
200 µm
300 µm

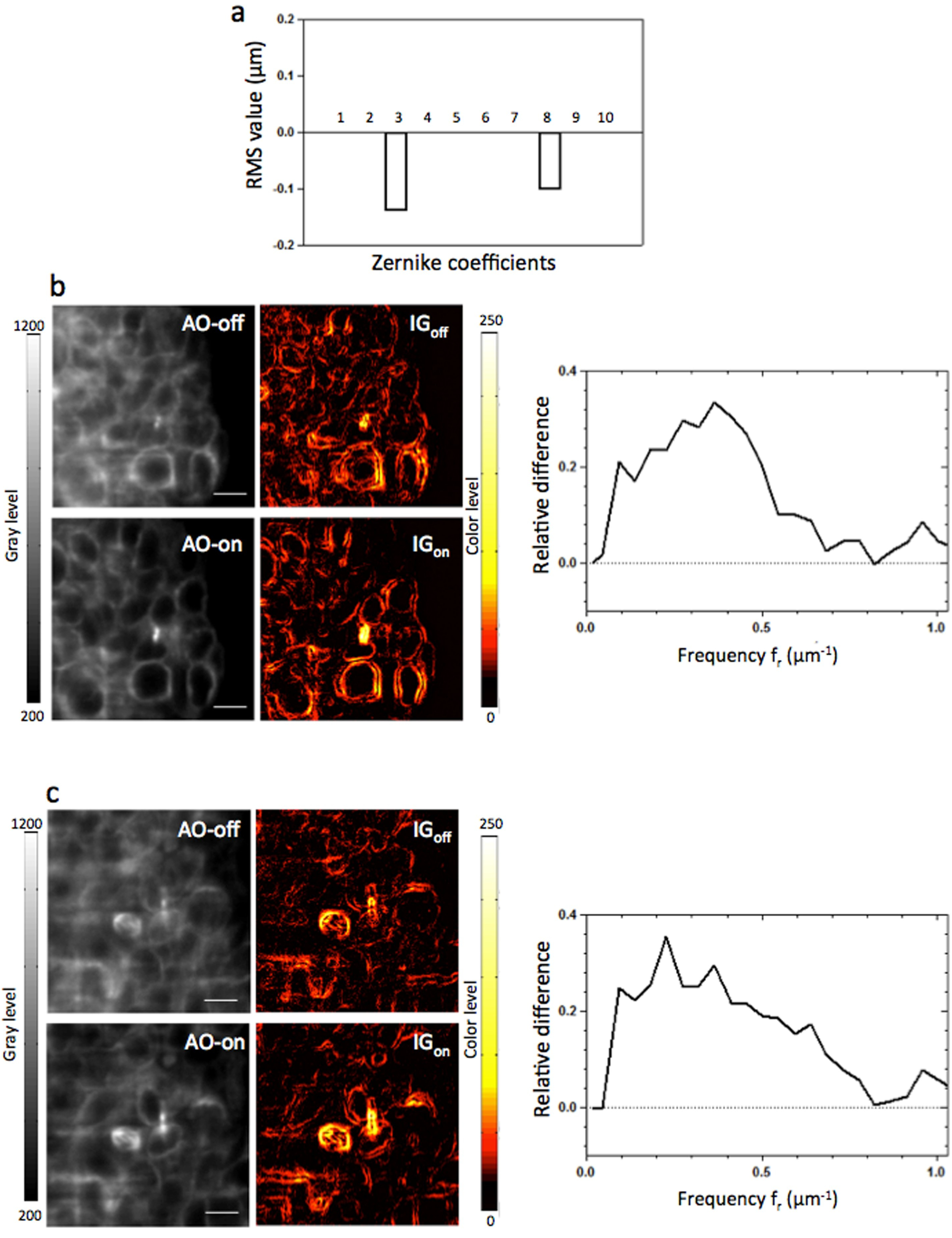
a
RMS value (µm)
0.2
0.1
0.0
-0.1
-0.2
1 2 3 4 5 6 7 8 9 10
Zernike coefficients
b
AO-off
AO-on
IG$_{off}$
IG$_{on}$
1200
200
Gray level
250
0
Color level
Relative difference
0.4
0.2
0.0
0.0 0.5 1.0
Frequency f_r (μm^{-1})
c
AO-off
AO-on
IG$_{off}$
IG$_{on}$
1200
200
Gray level
250
0
Color level
Relative difference
0.4
0.2
0.0
0.0 0.5 1.0
Frequency f_r (μm^{-1})

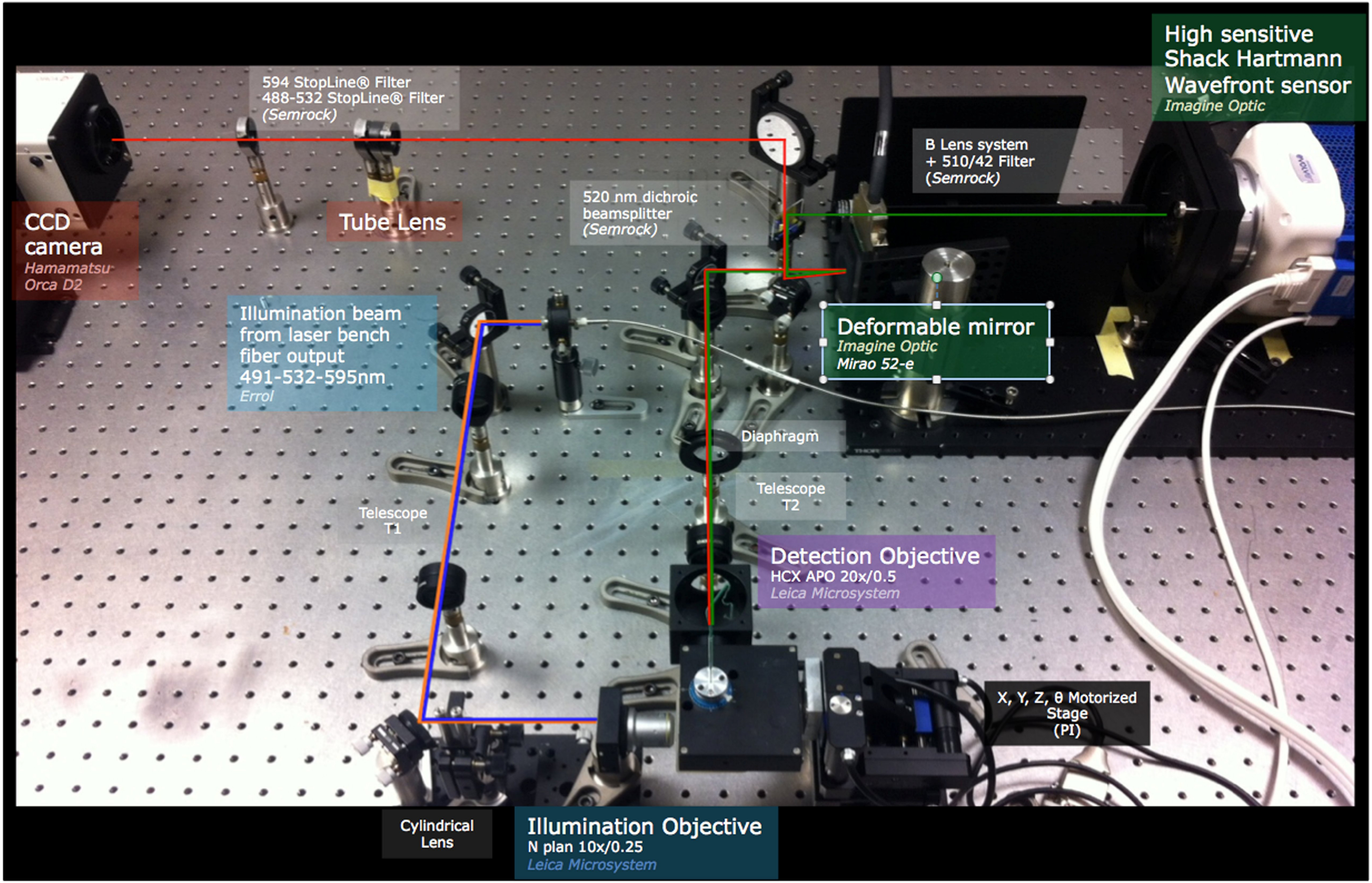
594 StopLine® Filter
488-532 StopLine® Filter
(Semrock)
High sensitive Shack Hartmann Wavefront sensor
Imagine Optic
B Lens system
+ 510/42 Filter
(Semrock)
520 nm dichroic beamsplitter
(Semrock)
CCD camera
Hamamatsu Orca D2
Tube Lens
Illumination beam from laser bench fiber output
491-532-595nm
Errol
Deformable mirror
Imagine Optic
Mirao 52-e
Diaphragm
Telescope T2
Telescope T1
Detection Objective
HCX APO 20x/0.5
Leica Microsystem
X, Y, Z, θ Motorized Stage
(PI)
Cylindrical Lens
Illumination Objective
N plan 10x/0.25
Leica Microsystem
